# Human neuroglial cells internalize *Borrelia burgdorferi* by coiling phagocytosis mediated by Daam1

**DOI:** 10.1371/journal.pone.0197413

**Published:** 2018-05-10

**Authors:** Shanna K. Williams, Zachary P. Weiner, Robert D. Gilmore

**Affiliations:** Bacterial Diseases Branch, Division of Vector Borne Diseases, National Center for Emerging and Zoonotic Infectious Diseases, Centers for Disease Control and Prevention, Fort Collins, Colorado, United States of America; University of North Dakota School of Medicine and Health Sciences, UNITED STATES

## Abstract

*Borrelia burgdorferi*, the agent of Lyme borreliosis, can elude hosts’ innate and adaptive immunity as part of the course of infection. The ability of *B*. *burgdorferi* to invade or be internalized by host cells in vitro has been proposed as a mechanism for the pathogen to evade immune responses or antimicrobials. We have previously shown that *B*. *burgdorferi* can be internalized by human neuroglial cells. In this study we demonstrate that these cells take up *B*. *burgdorferi* via coiling phagocytosis mediated by the formin, Daam1, a process similarly described for human macrophages. Following coincubation with glial cells, *B*. *burgdorferi* was enwrapped by Daam1-enriched coiling pseudopods. Coiling of *B*. *burgdorferi* was significantly reduced when neuroglial cells were pretreated with anti-Daam1 antibody indicating the requirement for Daam1 for borrelial phagocytosis. Confocal microscopy showed Daam1 colocalizing to the *B*. *burgdorferi* surface suggesting interaction with borrelial membrane protein(s). Using the yeast 2-hybrid system for identifying protein-protein binding, we found that the *B*. *burgdorferi* surface lipoprotein, BBA66, bound the FH2 subunit domain of Daam1. Recombinant proteins were used to validate binding by ELISA, pull-down, and co-immunoprecipitation. Evidence for native Daam1 and BBA66 interaction was suggested by colocalization of the proteins in the course of borrelial capture by the Daam1-enriched pseudopodia. Additionally, we found a striking reduction in coiling for a BBA66-deficient mutant strain compared to BBA66-expressing strains. These results show that coiling phagocytosis is a mechanism for borrelial internalization by neuroglial cells mediated by Daam1.

## Introduction

*Borrelia burgdorferi*, the bacterial agent of Lyme borreliosis, is a tick-borne pathogen of humans maintained in an enzootic cycle that encompasses reservoir hosts (commonly rodents) and *Ixodes spp*. ticks. *B*. *burgdorferi* exists as an obligate inhabitant of its tick vector and reservoir hosts, therefore the organism must adapt to the conditions encountered within these environments for survival. The spirochete accomplishes this adaptation through complex pathways of gene regulation to alter its protein synthesis profile (reviewed in [1]). Although the ecological cycle between ticks and mammalian hosts in nature is well established, molecular interactions that facilitate survival and virulence for *B*. *burgdorferi* within its hosts are only beginning to be uncovered.

In humans, infection is initiated by the deposition of *B*. *burgdorferi* into the skin via tick bite after which the spirochetes disseminate to colonize various organs and tissues causing manifestations affecting the joints (Lyme arthritis), heart (Lyme carditis), or nervous system (neuroborreliosis). Although susceptible to the host’s innate and adaptive immune responses, *B*. *burgdorferi* has proven to be adept at circumventing these defenses. Occupying the internal cellular niche has been suggested as a contributing factor in avoidance of clearance by antibodies or antimicrobials perhaps by providing an immune privileged refuge [2–4]. *B*. *burgdorferi*’s ability to invade or be internalized by human, murine, and tick cell lines in vitro and remain viable has been well documented [4–9]. This ability is facilitated by *B*. *burgdorferi* outer surface proteins that serve as adhesins allowing for attachment to various molecules of the extracellular matrix, cells, and serum thereby aiding in the establishment of tissue colonization and cellular invasion (reviewed in [10, 11]).

Internalization of *B*. *burgdorferi* also occurs via phagocytosis by cells of the innate immune system. Once deposited into the skin of humans by tick bite, *B*. *burgdorferi* encounters phagocytic and dendritic cells as the first line of host defense [12–15]. Internalization of *B*. *burgdorferi* by phagocytosis initiates a complex chain of trafficking pathways activating several cellular receptors that contribute to inflammation signaling largely accountable for the inflammatory responses seen in Lyme disease pathology (reviewed in [13, 16]).

Coiling phagocytosis is the predominant method of borrelial uptake whereby the macrophage envelopes the spiral shaped borrelia by elongated pseudopodia prior to internalization [17]. Cellular reorganization of the actin skeleton is required for formation of filopodia to begin the process of capturing borrelia, for the ensuing coiling of borrelia in pseudopodia, and for eventual cellular uptake [18]. Several actin-regulating proteins have been demonstrated to be critical in this process including actin-nucleating Arp2/3, the nucleation promoting WASP, and the formins FMNL1 and mDia [19, 20]. Recently described is disheveled-associated activator of morphogenesis (Daam1) as a regulator of *B*. *burgdorferi* phagocytosis by dually affecting filopodia regulation and pseudopodia formation [21]. Daam1 belongs to the formin family of proteins that have several functions including the nucleation and elongation of actin filaments in cells [22]. Although the cellular processes occurring during phagocytic uptake and processing within the cell are being uncovered, *B*. *burgdorferi* components that interact with cells to either initiate or evade phagocytosis are understudied. However, progress is being made as a recent investigation by Carrasco et al, associated the borrelial outer surface protein, OspC, as an anti-phagocytic factor against macrophage uptake [23].

Previously, we observed *B*. *burgdorferi* internalization of human neuroglial cells in vitro in a study to investigate borrelial invasion of cells of the central nervous system [7]. Our aim was to develop a model system to investigate mechanisms of borrelial cellular invasion and to identify protein-protein interactions that facilitate the process. In this study, we demonstrate that uptake of borrelia by neuroglial cells is accomplished by coiling phagocytosis and is mediated by colocalization with the formin Daam1. We also present evidence that the *B*. *burgdorferi* outer surface lipoprotein BBA66 binds to Daam1 at its formin homology-2 (FH2) domain.

## Materials and methods

### Bacterial strain and cell line culture conditions

Clonal populations of *B*. *burgdorferi* B31-A3-derived strains [24] were propagated to mid- to late-logarithmic stage in BSK-II media at 34°C in capped tubes providing microaerophilic conditions. Cultures were supplemented with kanamycin (200 μg/ml) and gentamicin (50 μg/ml) as required. A full complement of plasmids (except for cp9, which is missing in the B31-A3 strain) was demonstrated based on a multiplex PCR [25]. *B*. *burgdorferi* B31-A3 wild type (WT) and the B31-A3 mutant strain with inactivated *bba66* [26] expressing green fluorescent protein (GFP) were generated by introducing the *gfp*-containing plasmid, pMC2498 [27] (provided by Melissa Caimano, University of Connecticut), into the cells by electroporation as described [28]. B31-5A4NP1 strain [29] was engineered to produce BBA66 by fusing the constitutive *flaB* gene promoter to the *bba66* coding sequence in pMC2498 by replacing the *flaB* driven-*gfp* coding sequence with the *bba66* coding sequence (produced by PCR amplification) by EcoRI-BamHI digestion sites present downstream of the *flaB* promoter. Additionally, the *flaB-bba66* cassette from pMC2498 was excised by XbaI-BamHI digestion and subcloned into the multiple cloning site of pBSV2G, a vector used for transforming *B*. *burgdorferi* [30, 31]. The two *flaB-bba66* cassette plasmid constructs were separately electroporated into B31-5A4NP1 resulting in both cis- and trans-expressed *bba66* strains, B31:pMC2498-BBA66 and B31:pBSV2G-BBA66. The strains were induced for BBA66 production by growth in BSK-II pH 7.5 at 34°C in capped tubes to a density of 5 x 10^7^ cells per ml. 1 ml of each mid-logarithmic cultures was pelleted and resuspended in 4 ml of BSK-II media pH 6.8, and incubated at an elevated temperature (37°C) for 24 hrs. Cultures were grown under kanamycin and gentamicin selection. Multiplex PCR established that each BBA66-expressing *B*. *burgdorferi* strain contained all plasmids as the parental B31-5A4NP1.

Human neuroglioma cell lines, H4 and HS683, were purchased from the American Type Culture Collection (ATCC, Manassas, Virginia), maintained in 25 ml vented cell culture flasks (Corning, Corning, NY) and grown in Dulbecco’s modified Eagle’s medium (DMEM) with 4 mM l-glutamine containing 4.5 g/l glucose, 1.5 g/l sodium bicarbonate, and supplemented with 10% fetal bovine serum. All cells were grown at 37°C with 5% CO_2_ in a humidified incubator.

### *B*. *burgdorferi* cell infections and immunofluorescent staining

Cell coincubations with *B*. *burgdorferi* followed by immunofluorescent staining were performed as follows for all assays:

H4 and HS683 cells were grown to confluency, harvested using 0.05% trypsin with 10 mM EDTA, and enumerated using a Petroff-Hausser counting chamber. Cells were seeded at a density of 1 × 10^6^ cells / well in cell culture plates containing coverslips (Corning). Cells were allowed to attach to the coverslips for 24 hr and cell culture medium was replaced with fresh media prior to addition of *B*. *burgdorferi* culture. *B*. *burgdorferi* were added to the cells, and incubated at 37°C with 5% CO_2_. At each time point, coated coverslips were washed (3X) with Hanks Buffered Balanced Saline (HBSS) to clear unassociated spirochetes from the monolayers, followed by fixation. Coverslips were washed (3X HBSS) and stored in the dark at 4°C until further staining was performed. After fixation, the coverslips were blocked to prevent non-specific binding, washed (3X HBSS) prior to immunofluorescent staining.

For immunofluorescent stainings, all antibody incubations were followed by 3X HBSS washes. Cellular Daam1 was stained using rabbit polyclonal anti-Daam1 antibody (Proteintech, Rosemont, IL) (1:100) for 1 hr at 37°C in a humidified chamber, washed, and stained with anti-rabbit IgG Alexafluor 594 (Molecular Probes by Life Technologies, Carlsbad, CA) (1:100) for 1 hr at 37°C in a humidified chamber. Non-GFP *B*. *burgdorferi* strains were stained with fluorescein-labelled goat anti-*Borrelia burgdorferi* IgG (1:200) (Kirkegaard and Perry Laboratory Inc.) for 1 hr at 37°C in a humidified chamber. BBA66 was stained with mouse monoclonal anti-BBA66 antibody (1:100) for 1 hr, 37°C, washed, and stained with anti-mouse IgG Alexafluor 594 (Molecular Probes) (1:100) for 1 hr at 37°C in a humidified chamber. Anti-BBA66 monoclonal antibody (mouse IgG) was generated from rBBA66 immunization by the Antibody Purification and Development Team (Centers for Disease Control and Prevention Biotechnology Core Facility, Atlanta, GA).

Coverslips were washed (3X HBSS), and given a final water rinse to remove any residual salts. Coverslips were inverted and mounted with ProLong Diamond Antifade or ProLong Diamond Antifade with DAPI (Molecular Probes by Life Technologies) onto glass slides and placed at 4°C for 24 hr before being viewed by confocal microscopy. Samples were viewed using a Zeiss LSM 800 confocal laser scanning microscope (Carl Zeiss, Inc.). All images were analyzed using the LSM Software Zen 2 (Blue Edition). Colocalization quantification was performed for each image by intensity correlation analysis using the Coloc2 application in the Fiji ImageJ bundle (https://fiji.sc/). Colocalization is defined as the synchronous increase or decrease in fluorescence intensities as predicted when labeled proteins are part of the same molecular complex. In our analysis, the intensity correlation quotient (ICQ) was devised as a single coefficient to account for the covariance of mean deviation products in a defined area. The ICQ is calculated by the ratio of the number of positive values to the total number of pixels with mean deviation products. The ICQ is then corrected by subtracting 0.5 from the resulting ratio, resulting in a value corrected to a scale of -0.5 for no colocalization, 0.0 for randomness, and +0.5 for complete colocalization.

### Cell infection time course to image coiling of *B*. *burgdorferi*

Cell infection assays were performed in duplicate for each cell line and time point (technical replicate) and were repeated three times (biological replicate). The assays were performed as described above with the following modifications. Cells were seeded on laminin-coated coverslips (Corning Inc., Corning, NY) in 12 well culture plates. After cell attachment for 24 hr, GFP-expressing WT *B*. *burgdorferi* were added to the cells at a multiplicity of infection (MOI) of 10 with time points taken at 1, 4, 6, 8, and 12 hr post-infection. Cell fixation was performed with 4% paraformaldehyde at room temperature for 20 min. After fixation, the coverslips were incubated in phosphate buffered saline (PBS) with 2% bovine serum albumin (BSA) for 30 min to block non-specific binding. Cells were permeabilized by incubating the coverslips with 2% BSA and 0.5% Triton X-100 for 30 min at room temperature. Cells were stained for Daam1, coverslips mounted on glass slides and imaged by confocal microscopy. Samples were viewed using a Zeiss LSM 5 Pascal confocal laser scanning microscope. Images were analyzed using the LSM Software Zen 2009 (Carl Zeiss Inc.).

### Treatment with blocking antibodies against Daam1

Cell infection assays and immunofluorescent stainings were performed in duplicate for each cell line, timepoint, and treatment as described above with the following modifications. Cells were seeded in 6 well cell culture plates containing poly-L-lysine coated coverslips (Corning) for 24 hr. Cell culture medium was replaced with fresh media containing 10 μg rabbit polyclonal anti-Daam1 antibody (Proteintech) with incubation for 10 min followed by addition of GFP-expressing WT *B*. *burgdorferi* to the cells at a MOI of 100, and incubated at 37°C with 5% CO_2_. Cells were fixed with methanol at -20°C for 20 min after 1 and 2 hr post-infection. After fixation, coverslips were blocked (Pierce Protein Free T20 Blocking Buffer TBS) for 30 mins at room temperature. Cells were stained for Daam1, and mounted onto glass slides using ProLong Diamond Antifade with DAPI (Molecular Probes by Life Technologies). Slides were viewed using a Zeiss LSM 800 confocal laser scanning microscope to count the number of coiling events detected (Daam-1 pseudopods enwrapping a spirochete) in 1000 total neuroglial cells per coverslip.

### Cell infections and immunofluorescence labeling of BBA66-expressing *B*. *burgdorferi* and human Daam1

Cell infection assays and immunofluorescent stainings were performed in duplicate for each cell line, *B*. *burgdorferi* strain, and timepoint as described above with the following modifications. Neuroglial cells were infected with *B*. *burgdorferi* BBA66-expressing strains at a MOI of 100, followed by fixation with methanol at -20°C for 20 min after 1 and 2 hr post-infection. After coverslips were blocked, cells were incubated with rabbit anti-Daam1 antibody (Proteintech) followed by Pacific Blue goat anti-rabbit IgG (Molecular Probes by Life Technologies) (1:100) for 1 hr at 37°C in a humidified chamber. Cells were costained for BBA66 (as described above) and the *B*. *burgdorferi* strains B31:pBSV2G-BBA66 and B31:pMC2498-BBA66 were stained with fluorescein-labelled goat anti-*Borrelia burgdorferi* IgG (1:200) (Kirkegaard and Perry Laboratory Inc.) for 1 hr at 37°C in a humidified chamber. Colocalization calculations were performed as described above.

### Cell infections comparing coiling events between BBA66- expressing *B*. *burgdorferi* and BBA66-deficient *B*. *burgdorferi*

Cell infection assays and immunofluorescent stainings were performed in duplicate for each cell line, *B*. *burgdorferi* strain, and timepoint as described above with the following modifications. H4 and HS683 cells were grown and seeded on poly-L-lysine coverslips for 24 hr. *B*. *burgdorferi* B31:pMC2498-BBA66 and B31:pBSV2G-BBA66 were added to the cells at a MOI of 100. Cells were fixed with methanol at -20°C for 20 min after 1 and 2 hr post-infection. Cells were stained for Daam1 and *B*. *burgdorferi* strains B31:pBSV2G-BBA66 and B31:pMC2498-BBA66 were stained with fluorescein-labelled goat anti-*Borrelia burgdorferi* IgG (1:200) (Kirkegaard and Perry Laboratory Inc.) for 1 hr at 37°C. Coverslips were mounted with ProLong Diamond Antifade with DAPI (Molecular Probes by Life Technologies) and viewed using the Zeiss LSM 800 confocal laser scanning microscope to enumerate the number of coiling events (Daam-1 pseudopods enwrapping a spirochete) identified in 1000 total neuroglial cells.

### Detection of *B*. *burgdorferi* BBA66 by immunofluoresent staining

Induced *B*. *burgdorferi* B31:pMC2498-BBA66 and B31:pBSV2G-BBA66 were grown to a density of ~1 x 10^8^ cells / ml and 1 ml of each strain was collected, centrifuged, and washed (3X HBSS). Washed cells were resuspended in 100 μl of dH_2_O and spotted onto glass slides and allowed to air dry. Dried cells were fixed using methanol at -20°C for 20 min followed by 3X HBSS washes, and subsequent addition of blocking buffer (Pierce Protein Free T20 Blocking Buffer TBS, Pierce) for 30 mins at room temperature. Cells were washed and incubated with fluorescein-labelled goat anti-*Borrelia burgdorferi* IgG (1:200) (Kirkegaard and Perry Laboratory Inc. Gaitherburg, Maryland) for 1 hr at 37°C in a humidified chamber. 3X HBSS washes were performed followed by incubation with mouse monoclonal anti-BBA66 antibody (1:100) for 1 hr at 37 °C, washed (3X HBSS), and stained with anti-mouse IgG Alexafluor 594 (1:100) for 1 hr at 37°C. Final cell washes were performed (3X HBSS and 1X dH_2_O) and ProLong Diamond Antifade was used to mount coverslips onto glass slides and placed at 4°C for 24 hr. Samples were viewed using a Zeiss LSM 800 confocal laser scanning microscope. Images were analyzed using the LSM Software Zen 2 (Blue Edition) (Carl Zeiss Inc.).

### Yeast 2-hybrid (Y2H) library and bait clone construction

*Saccharomyces cerevisiae* yeast strains, Y2HGold and Y187 (Clontech Laboratories, Inc., Mountain View, CA) were cultivated in YPDA broth (Clontech), streaked, and stored on YPDA plates. Transformant bait and prey clones of Y2HGold and Y187 were cultivated in specialized broth and agar media purchased as premixed specialized pouches (Clontech). Yeast colonies were grown as appropriate on synthetically defined (SD) medium: SD base (supplies all nutrients needed for growth); SD/–Leu/–Trp (double) dropout supplement (selects for transformants containing bait and prey plasmids); SD/–Ade/–His/–Leu/–Trp (quadruple) dropout supplement (selects for the bait and prey plasmids, and for the activation of the Gal-responsive HIS3 and ADE2 genes as part of the confirmation step of the two-hybrid assay). Aureobasidin A was included as appropriate for antibiotic selection (Clontech).

The Y2H system, MatchmakerGold Yeast Two-Hybrid System (Clontech), was the source for reagents used for bait plasmid constructs, yeast transformation, and yeast mating. Detailed procedures were performed according to the manufacturer’s protocol manual (http://www.clontech.com/US/Products/Protein_Interactions_and_Profiling/Yeast_Two-Hybrid/ibcGetAttachment.jsp?cItemId=17597&fileId=6735138&sitex=10020:22372:US).

The BBA66 coding sequence was cloned in frame into the bait plasmid pGBKT7 to generate a GAL4 DNA-BD (binding domain) fusion. The BBA66 bait plasmid was transformed into the yeast strain, Y2H Gold, and transformants were selected by plating on SD/–Trp media. Yeast clones harboring pGBKT7-BBA66 were tested for auto-activation of reporter genes in the absence of a prey protein to eliminate false positive interactions. The BBA66 bait clone was tested for expression of BBA66 by Western blotting.

Y2HGold bait strains were mated with the Mate & Plate Library -(Normalized) Universal Human library as described in the manufacturer’s instructions (Clontech). Prey inserts from the library were cloned into the plasmid pGADT7 (Clontech). The mated yeast was plated on SD/–Ade/–His/–Leu/–Trp (quadruple) dropout media to select for activation of four reporter genes indicating expression and binding together of bait and prey proteins. Positive bait and prey protein interactions were observed as blue yeast colonies by the chromogen, X-α-gal, incorporated into the plates.

### Confirmation of positive bait and prey interactions in yeast

Phenotypic confirmation was performed by re-streaking blue colonies onto the high stringency quadruple dropout selective media containing X-α-gal (for colorimetric identification) to confirm growth. A single blue colony was picked and the prey plasmid was isolated using the Easy Yeast Plasmid Isolation Kit (Clontech) and transformed into *Escherichia coli* for propagation. *E*. *coli* transformants were plated on Luria-Bertani (LB) media supplemented with 250 μg / ml carbenicillin which selected for clones containing the prey plasmid containing the ampicillin resistance gene. Prey plasmid was isolated from selected *E*. *coli* transformants using Qiagen Mini-Prep Kit (Qiagen, Valencia, CA). The positive protein-protein binding interaction was confirmed by cotransforming the pGADT7-FH2/Daam1 prey plasmid and the pGBKT7-BBA66 bait into yeast strain Y2HGold. As the confirmatory controls, yeast strain Y2HGold was cotransformed with i) pGADT7-FH2/Daam1 prey plasmid and bait plasmid pGBKT7 with no cloned insert, or ii) pGADT7-FH2/Daam1 prey plasmid with pGBKT7 containing the *bba64* coding sequence.

### Sequence analysis of positive prey clone

The prey insert was identified by DNA sequence analysis from the prey plasmid, pGADT7, propagated in *E*. *coli* using the primer sets specific for the plasmid, Matchmaker AD LD-Insert Screening Amplimer Set (Clontech).

### Recombinant protein production

The BBA66 coding sequence minus the signal sequence and lipidation motif was amplified by PCR from *B*. *burgdorferi* B31 using primers designed for expression cloning into the pETite N-His vector according to the T7 Expresso system instructions (Lucigen, Middleton, WI) [32]. The constructed expression plasmids were transformed into *E*. *coli* 10G (Lucigen) and selected for growth on LB plates supplemented with 50 μg/ml kanamycin. DNA sequencing of the plasmid insert from transformants was performed to confirm the BBA66 coding sequence was correctly in frame for expression. Recombinant BBA66 pET-plasmids were purified by miniprep (Qiagen, Valencia, CA) and transformed into *E*. *coli* BL21(DE3) (Lucigen). Following transformant screening for the appropriate clones, colonies were grown in LB-kanamycin (50 μg/ml) broth, and recombinant protein expression was induced by the addition of IPTG (isopropyl-D-thiogalactopyranoside; 1 mM). Cells were harvested at late-log-phase growth, and recombinant protein was purified under nondenaturing conditions using a nickel-nitrilotriacetic acid (Ni-NTA) Fast Start His Tag affinity purification kit (Qiagen). Proteins were dialyzed into phosphate buffered saline (PBS, pH 8.0) and quantified by bicinchoninic acid (BCA) assay (Thermo-Fisher Scientific, Rockford, IL). Recombinant OspC was generated in same manner as described previously [32].

FH2/Daam1 recombinant protein from the Y2H gene fragment clone was produced and purified by the T7 Expresso system in the pETite N-His vector as described above. The fragment was PCR-amplified from the Y2H prey plasmid.

### rBBA66 and rFH2/Daam1 ELISA

Recombinant FH2/Daam1 (rFH2/Daam1) and control proteins were diluted with carbonate buffer (90 mM NaHCO3, 60 mM Na2CO3; pH 9.6) and bound to 96-well Immulon 2HB format plates (Thermo Scientific) (500 ng/well) overnight at 4°C. The plate wells were subjected to five washes with Tris-buffered saline–Tween 20 (TBS-T; 20 mM Tris, 140 mM NaCl, 2.7 mM KCl, 0.05% Tween 20 [pH 7.4]) using a BioTek 405 Select plate washer (BioTek, Winooski, VT), followed by addition of blocking buffer (300 μl) (Pierce Protein Free T20 Blocking Buffer TBS, Pierce, Rockland IL) for 1 hr at room temperature with agitation. Recombinant BBA66 (rBBA66) was labeled with biotin (EZ-Link Biotin No Weigh Kit, Pierce) according to the manufacturer’s directions and was used as a probe in the ELISA and pull-down assay. All incubations were performed for 1 hr at room temperature with agitation followed by washes (5X) in TBS-T. Biotin-labeled BBA66 was added to the protein-coated wells at 500 ng/well in TBS-blocking buffer, or at the designated concentrations in the dose dependent assays. After the wash cycle, alkaline phosphatase conjugated streptavidin (1:10,000) (Invitrogen) was added followed by the wash step. The reaction was stopped by adding 50 μl of 2 N NaOH to wells. Plates were read at an optical density at 405 nm (OD_405_) using an ELx808IU Ultra microplate reader (BioTek). Samples were assayed in triplicate with at least two separate technical runs.

### rBBA66 and rFH2/Daam1 pull-down assay

Biotin-labeled rBBA66 (9 μg) and rFH2/Daam1 (5 μg) were mixed together in 1.5 ml microfuge tubes on a HulaMixer (Invitrogen, Carlsbad, CA) in 200 μl PBS for 2 hr at room temperature. As controls, rFH2/Daam1 and biotin-rBBA66 were incubated independently in separate tubes. Pulldown columns were pre-tested with BSA and/other recombinant proteins prior to the experiments. The protein mixture was added to streptavidin-coated magnetic beads (Dynabeads M-280 streptavidin, Invitrogen) and allowed to mix for 2 hr at room temperature. The unbound supernatant was collected and the Dynabeads were washed (4X) in PBS. Proteins were eluted from the Dynabeads by the addition of 0.1% SDS and boiling for 5 min. The supernatant was collected, 2X sodium dodecyl sulfate polyacrylamide gel electrophoresis (SDS-PAGE) Laemmli loading buffer was added, and one half of the eluted sample was subjected to denaturing SDS-PAGE with fractionated proteins visualized by staining with GelCode Blue (Pierce). Following destaining, the gel was subjected to silver staining (Silver Stain Kit, Pierce) according to the manufacturer’s directions. The other half of the eluted sample was similarly subjected to SDS-PAGE followed by transfer to polyvinylidene difluoride (PVDF) membrane using the iBlot2 (Invitrogen) and immunoblotted against rabbit anti-Daam1 polyclonal antibody (1:2000) (Proteintech) according to standard procedures. The assay was repeated twice.

### rBBA66 and rFH2/Daam1 co-immunoprecipitation (CO-IP)

rFH2/Daam1 (10 μg) and rBBA66 (unlabeled, 10 μg) were mixed together in 1.5 ml microfuge tubes in 200 μl PBS overnight on the Hula Mixer at 4°C. As controls, rFH2/Daam1 and rBBA66 were incubated independently in separate tubes. Rabbit anti-Daam1 polyclonal antibody (1 μg) (Proteintech) was bound to Protein G conjugated magnetic beads (Dynabead Protein G, Invitrogen) according to the manufacturer’s directions. In a separate tube, mouse anti-BBA66 monoclonal antibody was bound to Dynabead Protein G. The protein mixtures were added to the antibody-coated Dynabeads and incubated with mixing (Hula Mixer) for 4 hr at room temperature. The unbound fraction was collected and the beads were washed 4X with Wash Buffer supplied with the Dynabead kit. Protein complexes were eluted from the beads by boiling in SDS-PAGE Laemmli loading buffer for 5 min followed by fractionation by SDS-PAGE. Gels were stained for protein visualization with GelCode Blue or transferred for immunoblot with anti-Daam1 and anti-BBA66 for band identification. The assay was repeated thrice.

## Results

### Human neuroglial cells H4 and HS683 internalize *B*. *burgdorferi* by coiling phagocytosis

**Fig 1:**
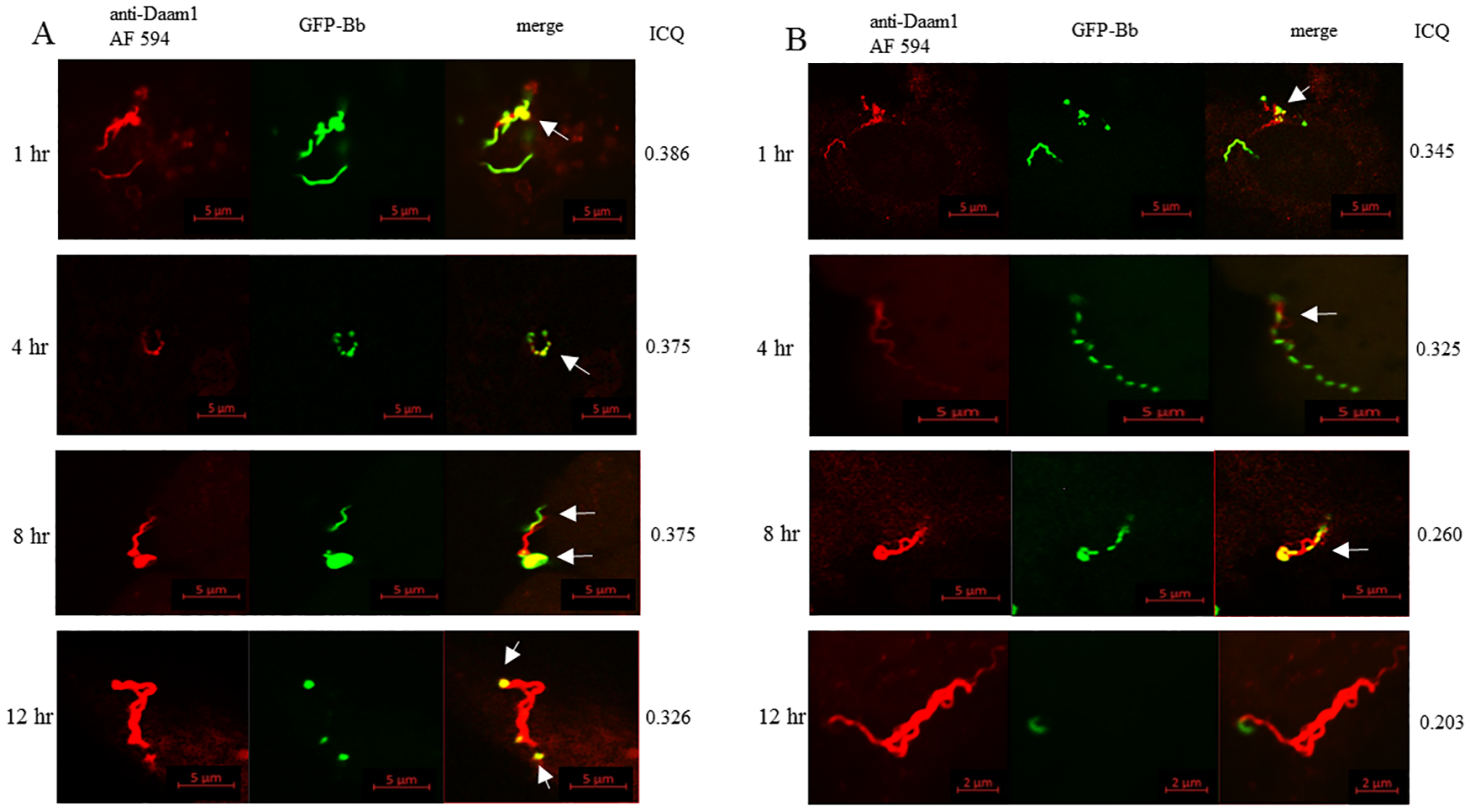
H4 and HS683 cells internalize *B*. *burgdorferi* by coiling phagocytosis. Coiling phagocytosis of *B*. *burgdorferi* by H4 and HS683 neuroglial cells viewed at 63X magnification after 1, 4, 8, and 12 hrs post-coincubation. A) H4 cells incubated with *B*. *burgdorferi*; B) HS683 incubated with *B*. *burgdorferi*. Fields are: left panel) anti-Daam1 stained with Alexafluor 594 (red); middle panel) GFP-*B*. *burgdorferi* (green); right panel) merged image. Note that after a few hours incubation, Daam1 enriched pseudopods engulf the spirochetes. Yellow in merged images indicate Daam1 colocalizing with *B*. *burgdorferi* outer membrane (arrows). ICQ values are denoted quantifying colocalization as described in Methods.

We incubated GFP-expressing *B*. *burgdorferi* with H4 and HS683 neuroglial cells and observed stages of coiling phagocytosis of *B*. *burgdorferi* by the cells during the 1, 4, 8, and 12 hours post-incubation periods (Fig 1). Immunofluorescent staining with anti-Daam1 antibody showed the collection of Daam1 along the pseudopodia contacting with the borrelial surface within 1 hr of co-incubation (Fig 1). Positive Daam1 immunostaining was observed for both permeabilized and non-permeabilized cells indicating the surface localization for Daam1 on the cell pseudopodia during coiling (data not shown). When the confocal images were merged between the Daam1 staining (red) and the *B*. *burgdorferi* (green), the yellow color indicated areas of colocalization between Daam1 and the organism. Colocalization quantification revealed ICQ values ranging from 0.203–0.386 for each cell line and time points, further validating the interaction between the spirochetes and the neuroglial cells (Fig 1). Incubation at 12 hr showed nearly the entire spirochete enveloped in Daam1-enriched pseudopods obscuring the green fluorescence of the organisms (Fig 1). This result demonstrates that the neuroglial cells recognize and begin *B*. *burgdorferi* coiling with Daam1 localizing to pseudopodia.

### Knockdown of phagocytosis by anti-Daam1 antibody treatment

The role of human Daam1 protein to mediate coiling phagocytic activity was examined by treating the H4 and HS683 cells with anti-Daam1 antibody prior to infection with GFP-expressing WT *B*. *burgdorferi*. At 1 and 2 hr coincubation times, coiling phagocytic events were assessed by counting the number of *B*. *burgdorferi* associations with Daam1-enriched pseudopodia per 1000 cells in the slide fields. Borrelia that fluorescently costained with Daam1 were counted to ensure that unattached spirochetes were not included as a coiling event.

**Fig 2:**
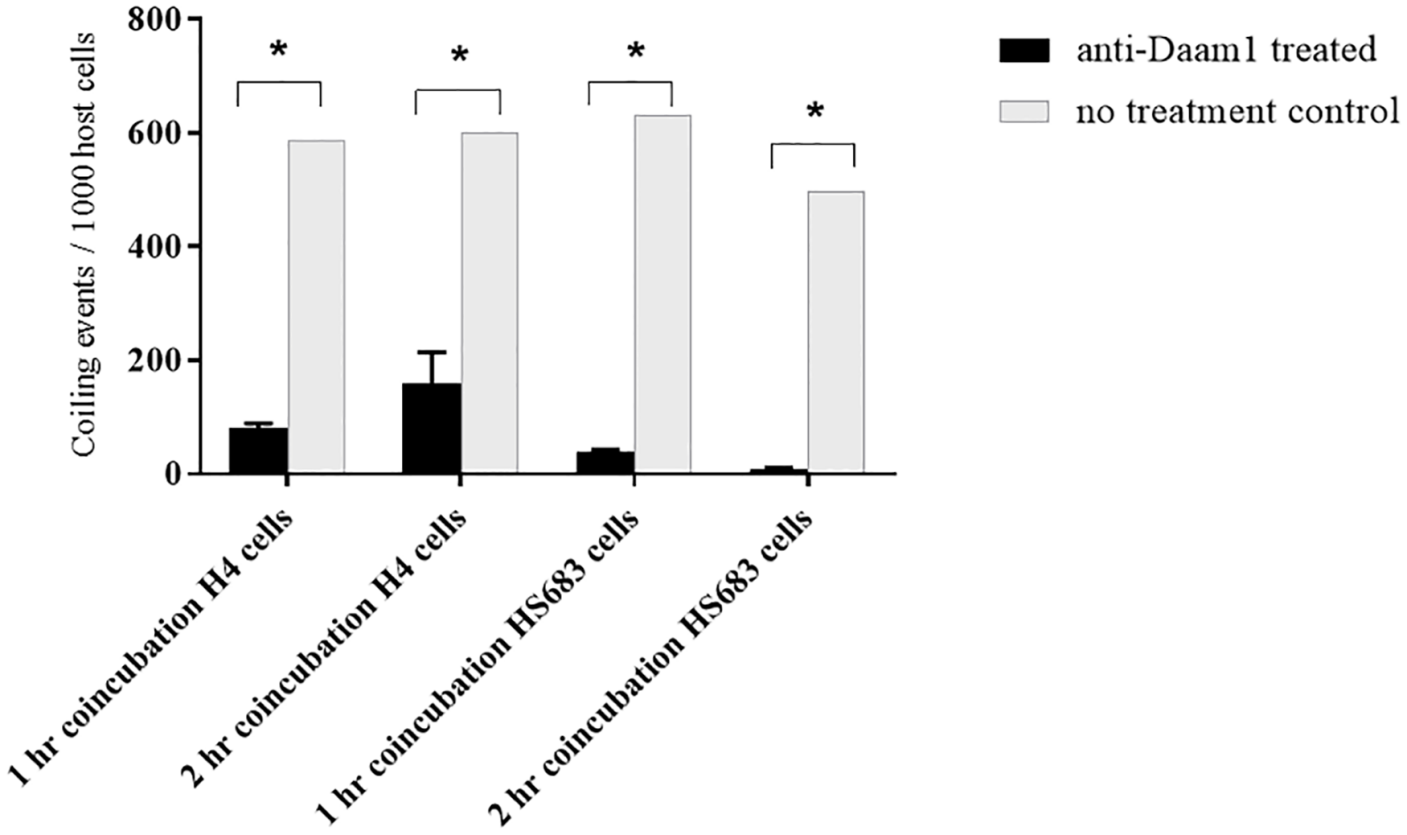
Anti-Daam1 antibody treatment of host cells reduces *B*. *burgdorferi* phagocytosis. Bars represent number of coiling events per 1000 host cells. Coiling event is defined as a host cell with a Daam1-stained pseudopod coiling around a *B*. *burgdorferi* cell. Asterisks denote P-value < 0.01 by Two-way ANOVA Sidak’s multiple comparisons test. Graphs represent the mean of all experiments.

The number of *B*. *burgdorferi* associated with coiling pseudopods was markedly reduced in cells treated with anti-Daam1 antibody compared to no antibody treated control cells. Both H4 and HS683 cell lines demonstrated similar results at both 1 and 2 hr incubation times (Fig 2). H4 treated cells showed 86% and 74% reduction in borrelial coiling events at 1 hr and 2 hr incubation respectively. HS683 treated cells showed 94% and 98.5% reduction in borrelial coiling events at 1 hr and 2 hr incubation respectively. This result is in accordance with the findings of Hoffman et al demonstrating Daam1 as a regulator for *B*.*burgdorferi* phagocytic uptake [21].

### Identification of Daam1 binding to *B*. *burgdorferi* BBA66 by yeast 2-hybrid (Y2H)

**Fig 3:**
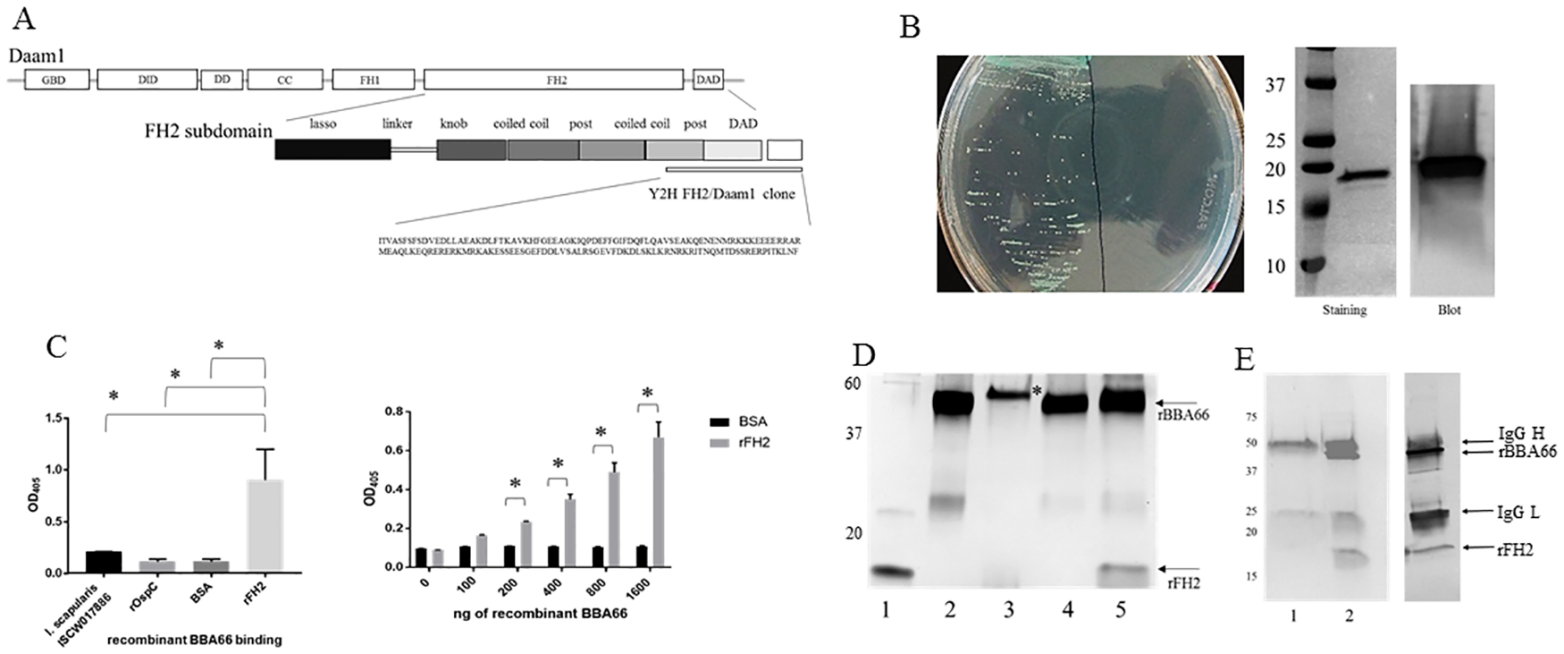
Recombinant BBA66 and FH2/Daam1 binding by Y2H confirmation and validating binding assays. A) Amino acid sequence and location of the Y2H Daam1-FH2 insert on the Daam1. Human Daam1 protein is illustrated at the top with the subdomains; GTPase binding domain (GBD); diaphanous inhibitory domain (DID); dimerization domain (DD); coiled coil (CC); formin-homology-1 (FH1); formin-homology-2 (FH2); diaphanous auto-regulatory domain (DAD). The FH2 subdomains are shown based on the crystal structure [33, 34]. B) left panel: Y2HGold yeast cotransformed with both BBA66-pGBKT7 and Daam1/FH2-pGADT7 and plated on quadruple dropout media (left side of plate). Y2HGold yeast cotransformed with control plasmid BBA64-pGBKT7 and Daam1/FH2-pGADT7 (right side of plate); right panel: Recombinant Y2H Daam1/FH2 on SDS-PAGE stained with GelCode Blue, and Recombinant Y2H Daam1/FH2 immunoblot with anti-Daam1. Molecular weight markers are denoted on the left in kilodaltons. C) Binding profiles by ELISA. (left) Recombinant BBA66 binding to antigens at 500 ng protein/well, p value determined by one-way ANOVA; (right) dose dependent binding of recombinant BBA66 to recombinant FH2/Daam1 and BSA control, p value determined by two-way ANOVA. * denotes p value < 0.001. D) Pull down assay binding of BBA66 and FH2/Daam1 by silver stained SDS-PAGE. Lanes: 1) purified recombinant FH2/Daam1; 2) purified recombinant BBA66; 3–5) elution fraction of pull down, 3) FH2/Daam1 only; 4) BBA66 only; 5) FH2/Daam1 and BBA66. Equal concentrations of protein were subjected to each pull down reaction and loaded in each lane. Arrows denote position of rBBA66, rFH2/Daam1. Molecular weight markers are denoted to the left. Asterisk in lane 3 denotes the 60 kDa subunit of streptavidin that eluted from the beads. E) CO-IP binding of FH2/Daam1 and BBA66 complex to anti-BBA66 antibody. Left) GelCode Blue staining; Lanes: 1) FH2/Daam1 only elution from protein G beads; 2) FH2/Daam1 and BBA66 elution from protein G beads. Right) Immunoblot probed with both anti-Daam1 and anti-BBA66 to identify bands in left panel, lane 2. Arrows indicate IgG heavy chain (50 kDa), BBA66 (45 kDa), IgG light chain (25 kDa), and FH2/Daam1 (17 kDa). Heavy and light chain appear on blot due to reaction with secondary alkaline-phosphatase conjugated anti-IgG.

Screening a Y2H human library with selected *B*. *burgdorferi* bait genes revealed a protein-protein interaction between BBA66 and the formin homology-2 (FH2) domain of Daam1. The Y2H FH2/Daam1 cloned insert consisted of 1002 bp with the first 441 bp in frame for protein expression ending in a TAG stop codon. The sequence aligned with the C-terminal fragment of the FH2 domain of human Daam1 (Fig 3A). The crystal structure of the human Daam1 FH2 domain has been reported and shows the Y2H fragment mapping to the S and T coiled-coil and post subdomains and extending into the C-terminal diaphanous auto-regulatory domain (DAD) of Daam1 [33, 34].

### Confirmation of FH2/Daam1 and BBA66 interaction by cotransformation in yeast

We confirmed the observed binding interaction from the initial screen of the human library as a true positive by cotransforming the BBA66 encoding gene bait plasmid and FH2/Daam1 prey plasmid into the yeast strain Y2HGold with subsequent plating on selective media. As a control, the FH2/Daam1 prey plasmid was cotransformed with the bait plasmid containing no insert. Blue-colored yeast transformant colonies containing the BBA66 and FH2/Daam1 plasmids were present following incubation on quadruple dropout media indicating that the binding of the expressed proteins activated the four reporter genes enabling growth and reaction with the chromogenic substrate (Fig 3B). No colony growth was recorded from the FH2/Daam1 prey and empty bait plasmid cotransformation. An additional control was performed whereby a bait plasmid containing the BBA64 protein encoding gene was cotransformed into Y2HGold with the FH2/Daam1 prey plasmid. This cotransformation also resulted in no colony growth on quadruple dropout selective media (Fig 3B).

### Validation of FH2/Daam1 and BBA66 binding by in vitro assays

*E*. *coli*-expressed recombinant (r)FH2/Daam1 was purified from the soluble lysate fraction to allow for possible conformational folding that could be eliminated by denaturing purification. rFH2/Daam1 migrated as a 17 kDa band on SDS-PAGE and was used for subsequent protein binding validation assays (Fig 3B). Rabbit anti-human Daam1 polyclonal antibody purchased commercially was reactive by immunoblot against rFH2/Daam1 and was used for protein-protein binding assays when required (Fig 3B).

rBBA66 binding to rFH2/Daam1 was performed by ELISA. Binding was first assessed by coating ELISA plate wells with a single concentration (500 ng) of rFH2/Daam1 and three control proteins not known to bind BBA66. rBBA66 bound rFH2/Daam1 significantly higher than the control proteins, *B*. *burgdorferi* OspC, *I*. *scapularis* ISCW017886, and bovine serum albumin (BSA) (Fig 3C). Further ELISA analysis found rBBA66 binding rFH2/Daam1 in a dose dependent manner compared to BSA control (Fig 3C).

rBBA66 and rFH2/Daam1 binding was evaluated by protein-protein pull-down assay. rFH2/Daam1 and biotin-labeled rBBA66 were incubated together and the complex was pulled down on streptavidin-conjugated magnetic beads. The eluted fraction was observed by SDS-PAGE silver staining and revealed rFH2/Daam1 bound to rBBA66 (Fig 3D, lane 5). As controls, biotin-labeled rBBA66 only bound to the streptavidin beads (Fig 3D, lane 4), while rFH2/Daam1 only was not bound indicating that this protein did not non-specifically bind to the streptavidin beads (Fig 3D, lane 3).

Protein-protein binding of rFH2/Daam1 and rBBA66 was similarly demonstrated by co-immunoprecipitation (CO-IP). The rFH2/Daam1-rBBA66 complex was precipitated by anti-BBA66 monoclonal antibody (Fig 3E, lane 2). The control of rFH2/Daam1 alone did not bind to the anti-BBA66 antibody demonstrating the specificity of the assay (Fig 3E, lane 1). Immunoblotting of the immunoprecipitated complex with anti-Daam1 and anti-BBA66 confirmed the band identities seen by GelCode Blue staining (Fig 3E). Identical results were also obtained when the rFH2/Daam1-rBBA66 complex was precipitated by the anti-Daam1 polyclonal antibody (data not shown). These results substantiate the finding of the Y2H assay that BBA66 and the FH2 subdomain of Daam1 are binding partners.

### Daam1-BBA66 colocalization during host cell infection

**Fig 4:**
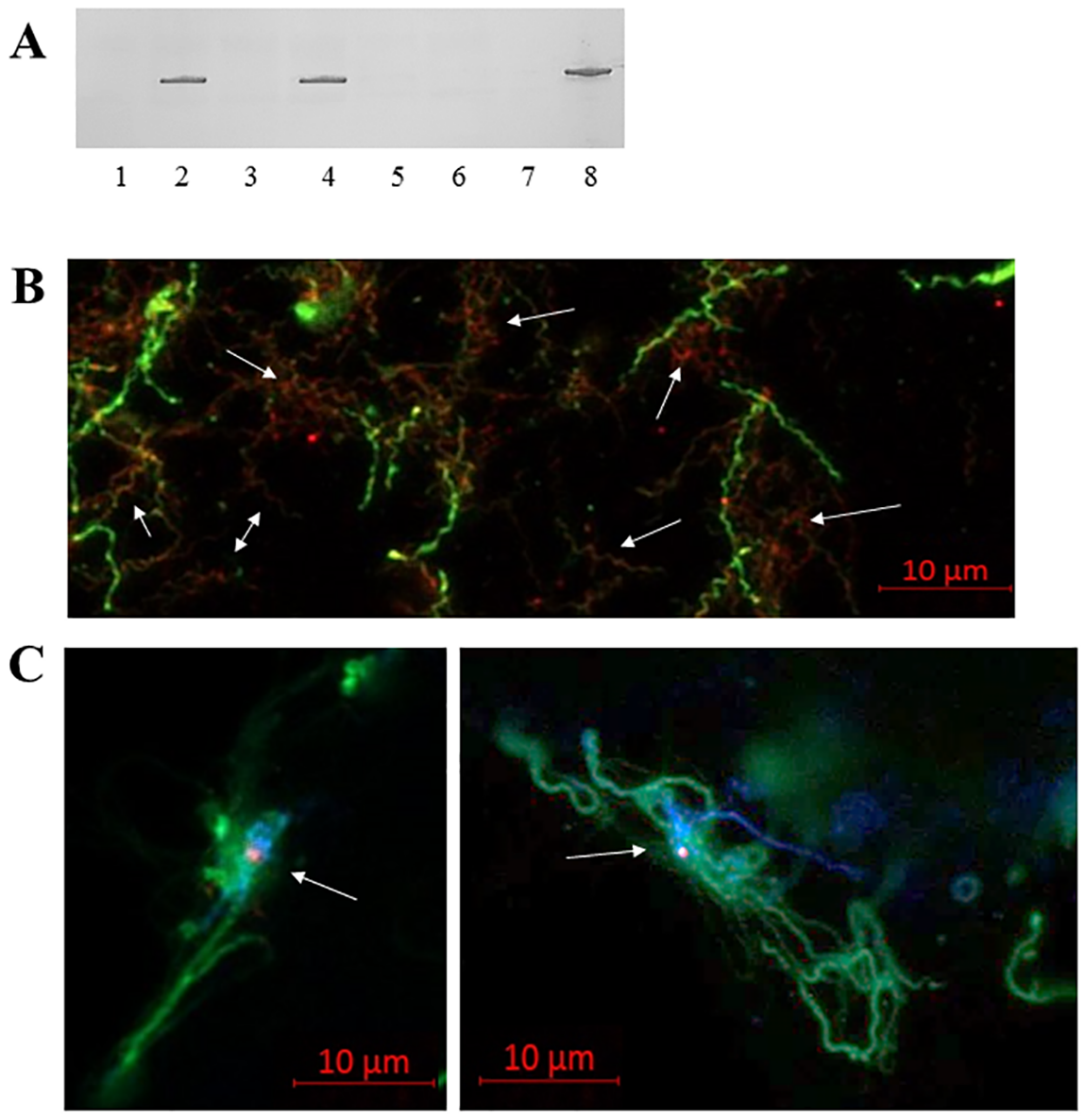
Colocalization of BBA66 to Daam1 during neuroglial cell infection. A) Western blot demonstrating BBA66 production by the BBA66-expressing *B*. *burgdorferi* strains. Lanes: 1) B31:pBSV2G-BBA66 pre- induced; 2) B31:pBSV2G-BBA66 induced; 3) B31:pMC2498-BBA66 pre induced; 4) B31:pMC2498-BBA66 induced; 5) B31-A3 WT grown at 34°C; 6) B31-A3 WT grown at 37°C; 7) BBA66 knockout mutant strain; 8) recombinant BBA66. B) IFA of induced B31:pMC2498-BBA66. *B*. *burgdorferi* are stained green and BBA66 stained red. Arrows indicate subpopulations of cells expressing BBA66 that appear with red punctate on green background. C) Colocalization of Daam1 to BBA66 during host H4 cell infection. *B*. *burgdorferi* stained with fluorescein (green), BBA66 stained with Alexafluor 594 (red), and Daam1 stained with Pacific Blue (blue) in the psuedopodia. Arrow indicates Daam1-BBA66 area of colocalization by merged blue and red color.

We showed that binding of BBA66 and Daam1/FH2 occurred with recombinant proteins, therefore our next aim was to demonstrate binding of the native proteins by *B*. *burgdorferi* cell infections in vitro. In our hands, *B*. *burgdorferi* B31-A3 produced little to no detectable BBA66 when grown in culture even when conditions were optimized for pH and temperature as other investigators have shown [35, 36]. To alleviate this issue, we generated *B*. *burgdorferi* clonal isolates to produce BBA66 by fusing the *bba66* coding sequence to the flagellin (*flaB*) gene’s constitutive promoter. Two separate plasmid backbone vectors were used for the construction, pBSV2G and pMC2498, that when transformed into *B*. *burgdorferi* resulted in a cis- and trans-integrated gene fusion. The newly isolated clones produced detectable BBA66 by immunoblot following a culture condition shift to pH 6.8 and 37°C (Fig 4A). BBA66 expression was also detectable by immunofluorescence (Fig 4B). Induced culture grown BBA66-expressing *B*. *burgdorferi* were coincubated with neuroglial cells and triple-stained following fixation. Fig 4C shows apparent colocalization of BBA66 with Daam1 in the pseudopod coiling around the borrelia.

### *B*. *burgdorferi* BBA66 knockout mutant strain demonstrates reduced uptake by coiling phagocytosis

**Fig 5:**
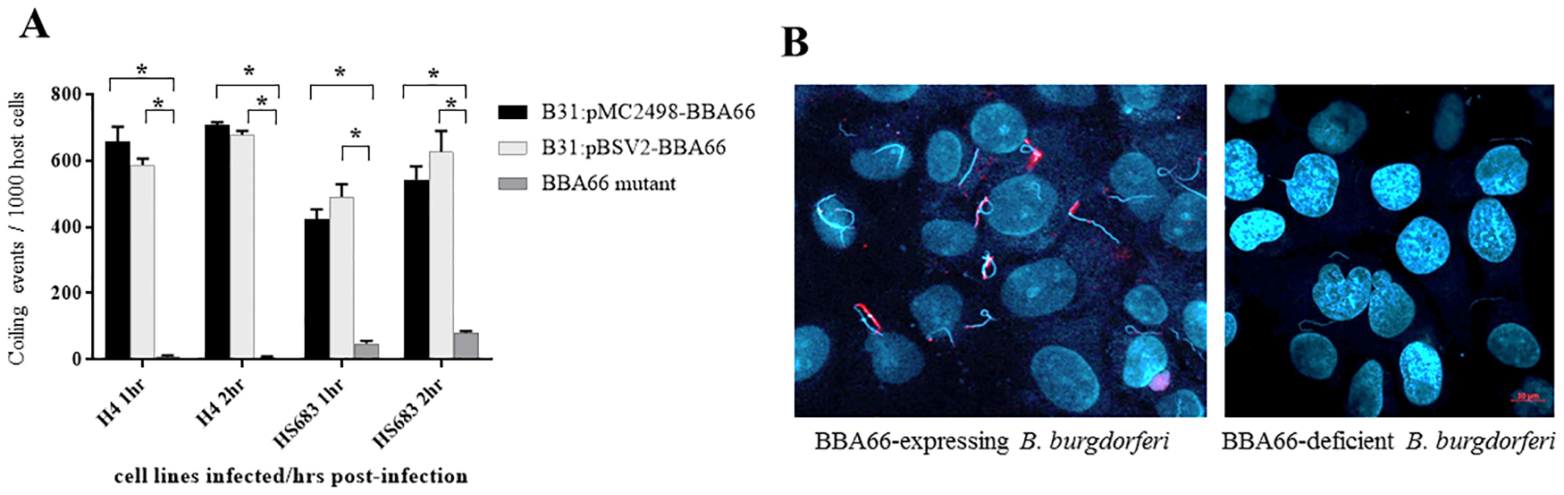
BBA66 knockout mutant strain demonstrates reduced coiling phagocytosis compared to BBA66-expressing strains. A) Bars represent number of coiling events per 1000 host cells. Coiling event is defined as a host cell with a Daam1-stained pseudopod coiling around a *B*. *burgdorferi* cell. Asterisks denote P-value < 0.001 by Two-way ANOVA Tukeys’s multiple comparisons test. Graphs represent the mean of all experiments. B) Representative field of H4 cell infection at 2 hrs post-incubation with B31:pMC2498-BBA66 (left panel) and with BBA66 knockout mutant strain (right panel). Daam1 stained red and *B*. *burgdorferi* and host cell nucleus stain blue by DAPI.

We performed additional cell infection assays with the two BBA66-expressing strains and a BBA66 knockout mutant strain to assess whether BBA66 had an effect on coiling phagocytosis. The BBA66 mutant was described in a previous report as having an attenuated tick-to-mouse infection defect [26]. After coincubation with H4 neuroglial cells for 1 and 2 hrs, both BBA66-expressing strains demonstrated similar coiling uptake (range of 587–708 coiling events/1000 cells) (Fig 5A), however the BBA66-deficient strain had a 99% reduction in Daam1-mediated coiling (Fig 5A). Similar results were seen in HS683 coincubations, whereby the BBA66-expressing strains ranged from 423–687 coiling events/1000 cells, with the BBA66-deficient mutant strain demonstrating an 85–90% reduction in coiling events (Fig 5A). Representative fields of cells coincubated with both BBA66-expressing and –deficient strains that were used for counting are shown in Fig 5B.

## Discussion

The purpose of this study was to continue our investigation into *B*. *burgdorferi* internalization of human cells, using the neuroglial model system for borrelial invasion we previously described [7]. In that study, we observed that *B*. *burgdorferi* was capable of associating with and being internalized by human H4 and HS683 cells.

Previously we observed the internalization of *B*. *burgdorferi* by neuroglial cells following 20 hours of coincubation but did not investigate earlier timepoints of cellular interaction to determine the mechanism of invasion [7]. Here, we observed *B*. *burgdorferi* interactions with host cells at earlier times, from 1–12 hours post-infection, and found that the neuroglial cells internalized the spirochetes by coiling phagocytosis in a manner similar to that described with human primary macrophages [21]. In our previous study, we did not observe cytopathic effects of H4 or HS683 cells following internalization of *B*. *burgdorferi* after 7 days post-infection, and we determined that the internalized borrelia remained viable following the approximate 20 hour incubation period [7]. Additional studies will be required to determine more information about long term survival of *B*. *burgdorferi* in these neuroglial phagocytes or whether they eventually submit to eventual destruction following uptake as reported for macrophages [12, 15, 37]. In vitro studies performed by Wu et al., using non-immune primary human dermal fibroblasts, reported *B*. *burgdorferi* remained viable up to 28 days following uptake [4]. Therefore, it remains to be elucidated whether *B*. *burgdorferi* are eventually destroyed by internalization of microglial cells or able to manufacture an intracellular survival mechanism.

Previous investigations have shown that *B*. *burgdorferi* stimulate the formation of filopodia followed by elongation of coiling pseudopods in primary human macrophages to effect the phagocytic uptake of the organism for intracellular processing [19, 20]. Specifically, Hoffman et al identified Daam1 as both a key regulator of actin polymerization to produce filopodia that capture the spirochete and as a component that accumulates and localizes to the pseudopodia that enwrap the borrelial cells [21]. Daam1 belongs to the formin family of proteins that have several functions including the nucleation and elongation of actin filaments in cells [22]. The formin protein family characteristically shares two common domains termed formin-homology 1 (FH1) and formin-homology 2 (FH2). FH2 binds actin and functions to nucleate and elongate actin filaments by associating with the barbed ends [38].

Prior to cell infection, Daam1 is present in the cytoplasm. Following *B*. *burgdorferi* stimulation, Daam1 accumulated and localized to the surface of the glial cells along the length of pseudopodia where it interacted with the borrelia. This surface localization for Daam1 on the pseudopodia was also reported for human macrophages [21]. This result demonstrates that human neuroglial cells produce Daam1 as part of the regulatory machinery for actin cytoskeleton polymerization required for *B*. *burgdorferi* coiling uptake. Treating the neuroglial cells with anti-Daam1 antibody prior to coincubation with *B*. *burgdorferi* resulted in a significant reduction of borrelial coiling indicating the requirement for Daam1 to mediate this process. This requirement for Daam1 was similarly demonstrated by the Linder laboratory study using silencing RNA whereby the knockdown of Daam1 expression reduced borrelial uptake [21].

Colocalization between *B*. *burgdorferi* and Daam1 in the neuroglial cell coiling pseudopodia suggested interaction with borrelial outer membrane protein(s). To identify potential host binding partners, we utilized a Y2H system library of human expressed gene products and found that the FH2 subunit domain of Daam1 bound the *B*. *burgdorferi* surface lipoprotein BBA66. Previously, we identified BBA66 as a factor enabling the spirochete to infect mice via tick bite transmission, but the functional aspects driving this process have not been defined [26]. The Y2H finding suggested a role for BBA66 in coiling phagocytosis for internalizing *B*. *burgdorferi*.

Several strategies were employed to confirm and validate the BBA66-FH2/Daam1 binding by the Y2H. First, cloned plasmids containing coding sequences for bait BBA66 and the prey FH2/Daam1 were cotransformed into yeast resulting in growth on highly selective media confirming the original interaction. Control transformations with either the prey plasmid with no insert, or prey plasmid containing the BBA64 coding sequence, demonstrated no yeast colony growth on selective media indicating that the FH2/Daam1 did not autoactivate the selection markers. Second, ELISA, pull-down, and CO-IP, demonstrated positive binding activities with recombinant forms of BBA66 and FH2/Daam1, providing further evidence for specific protein-protein binding.

The in vitro assays validated the Y2H binding, therefore we proceeded to cell culture models to explore whether native BBA66 and Daam1 proteins interacted in the course of *B*. *burgdorferi* envelopment by pseudopodia. Since limited BBA66 protein was made by BSK-cultured organisms, we constructed *B*. *burgdorferi* strains to express detectable BBA66 by fusing the constitutive *flaB* gene promoter to the *bba66* coding sequence. When using the BBA66-expressing strains in neuroglial cell infections, we observed apparent colocalization between BBA66 and Daam1 when borrelia were being captured by pseudopodia. Although colocalization was observed, co-immunoprecipitation to detect binding was inconclusive, therefore we employed another approach to observe whether BBA66 was involved in coiling by coincubating cells with BBA66 -expressing and –deficient strains. Interestingly, we found significantly higher numbers of BBA66-expressing borrelia being coiled compared to the BBA66-deficient mutant strain which suggested that Daam1-enriched pseudopodia may recognize BBA66 to capture borrelial cells. This observation suggests that expression of BBA66 would lead to the spirochetes’ internalization (with an eventual fate yet to be elucidated within neuroglial cells), whereas subpopulations of borrelia that downregulate BBA66 production may evade the phagocyte. However, we noted the WT strain contributed an equivalent number of coiling events when coincubated with the cells as did the BBA66- expressing strains (Figs 2 and 5) which was unusual considering that we did not detect WT BBA66 by immunoblot. Although we are unclear regarding the reason for this discrepancy, an explanation may lie in that low level expression of *bba66* and production of BBA66 in culture conditions has been demonstrated, suggesting that limited amounts of BBA66 are sufficient for cell phagocytic recognition [36, 39]. Although poorly expressed when grown in BSK culture, we previously found that *bba66* is upregulated in ticks during the course of bloodmeal acquisition, perhaps as a response required for borrelial tick transmission. Therefore, BBA66 is likely present on borrelia when deposited in the skin [26]. We have also observed differential *bba66* expression in tissues from experimentally infected mice suggesting borrelial adaptation to changing environments such as immune pressure or colonization [40, 41]. Overall, the experiments performed provide evidence for BBA66 binding to the Daam1 subunit, but the functional involvement for borrelial coiling remains unresolved.

## Conclusions

We previously demonstrated that *B*. *burgdorferi* were internalized by human neuroglial cells [7]. In this study we found that uptake occurs by coiling phagocytosis mediated by Daam1 localized to the pseudopodia that enwrap borrelia. However more work remains to resolve unanswered questions regarding borrelial host cell internalization, e.g. i) do neuroglia utilize the same or similar pathways to internalize and process *B*. *burgdorferi* as macrophages recruited to the skin tick bite site? ii) what are the pathways utilized for invasion / internalization of borrelia into non-phagocytes that differ from phagocytic cells? iii) is BBA66 or other surface components of *B*. *burgdorferi* recognized by Daam1 as part of the mechanism for pseudpodia envelopment? Adherence and invasion of host cells are drivers of infection by microbial pathogens. A comprehensive understanding of the molecular processes involved in this phase of *B*. *burgdorferi* pathogenesis are only beginning to be revealed with several aspects remaining to be explored.
